# Changes in North Atlantic Oscillation drove Population Migrations and the Collapse of the Western Roman Empire

**DOI:** 10.1038/s41598-017-01289-z

**Published:** 2017-04-27

**Authors:** B. Lee Drake

**Affiliations:** Department of Anthropology, University of New Mexico, MSC01-1040, Anthropology 1, Albuquerque, NM 87131 USA

## Abstract

Shifts in the North Atlantic Oscillation (NAO) from 1–2 to 0–1 in four episodes increased droughts on the Roman Empire’s periphery and created push factors for migrations. These climatic events are associated with the movements of the Cimbri and Teutones from 113–101 B.C., the Marcomanni and Quadi from 164 to 180 A.D., the Goths in 376 A.D., and the broad population movements of the Migration Period from 500 to 600 A.D. Weakening of the NAO in the instrumental record of the NAO have been associated with a shift to drought in the areas of origin for the Cimbri, Quadi, Visigoths, Ostrogoths, Huns, and Slavs. While other climate indices indicate deteriorating climate after 200 A.D. and cooler conditions after 500 A.D., the NAO may indicate a specific cause for the punctuated history of migrations in Late Antiquity. Periodic weakening of the NAO caused drought in the regions of origin for tribes in antiquity, and may have created a powerful push factor for human migration. While climate change is frequently considered as a threat to sustainability, its role as a conflict amplifier in history may be one of its largest impacts on populations.

## Introduction

At its height, the Roman Empire controlled a region including modern-day England, the southern half of Continental Europe, West Africa, and the Middle East. It contained over 20% of the world’s population and covered 5 million square kilometers. As a political unit Rome lasted from 600 BC to 410 A.D., and even surviving as far as 1453 A.D. if one considers the Byzantine Empire. The decline of the Roman Empire took place in the context of large population migrations in Europe, which occurred in two phases. The first phase began in 376 A.D. with the movement of Gothic tribes in response to Hunnic migration from Central Asia. The inability to control this migration led to the collapse of authority in the Western Roman Empire. Successive waves of migration by the Vandals, Alemani, Franks, Alans, and Goths overwhelmed the Western Roman Empire. The second wave of migrations would include Slavic-speaking and Turkic- speaking peoples, permanently altering the linguistic landscape of Eastern Europe (Supplemental Historical Material)^1^.

### Historical Background

**Figure 1:**
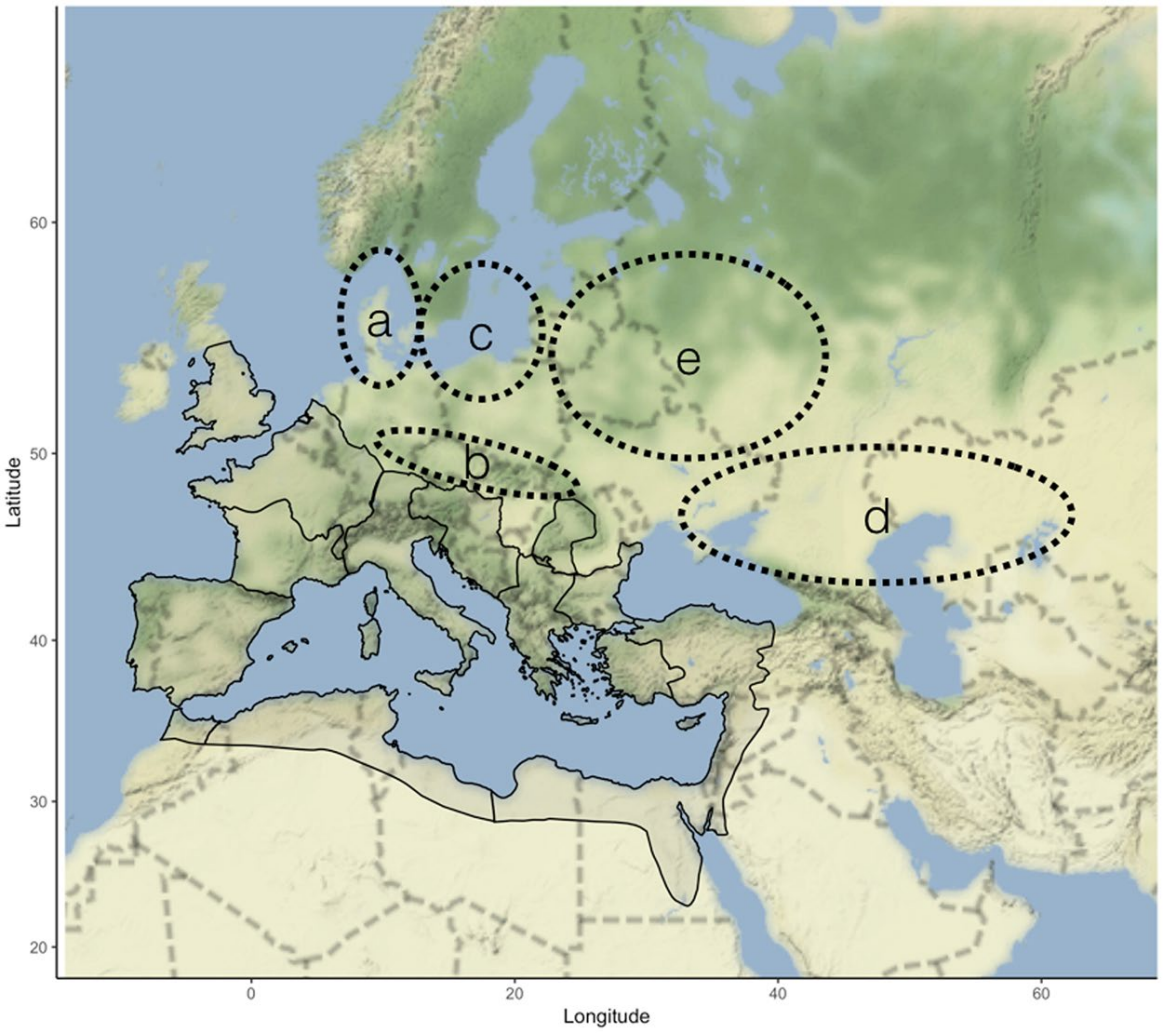
Roman Empire at its territorial height in 117 A.D. Locations are highlighted for (**a**) the Cimbri and Teutones before 117 B.C., (**b**) the Marcomanni and Quadi before 160 A.D., (**c**) hypothesized location for tribes which would eventually become the Goths before 370 A.D., (**d**) possible migration path of the Huns around 400 A.D., and (**e**) Slavic-speaking groups prior to the Migration Period (500 A.D.). There is low certainty in the placement of linguistic groups prior to the Migration Period. While linguistic territories were broad, populations were likely concentrated in smaller areas. Map generated in R (3.3.2)^40^ using map tiles by Stamen Design (under CC BY 3.0. Data by OpenStreetMap, under ODbL).

There were four significant proto-Germanic/Germanic migration events into territories associated with Roman Republic/Empire (Fig. 1):

### Cimbri migration (113–101 B.C.)

Event: The Cimbri and Teutones migrate from the Jutland Peninsula to northern Italy, defeating Roman legions in 112 B.C., 109 B.C., and 105 B.C. The Romans, under Gaius Marius (157–86 A.D.) reorganized the legions^2^ and coordinated a response to the migrations. Marius led the successful final campaign against the Cimbri and Teutones in 101 B.C.^3^.

Effects: The reorganization of the legions opened military service to the poor and provided a route to financial/social advancement^2^ through service to generals. Within years, generals marched on Rome to seize power. The resulting instability eroded democratic norms until power was centralized under Gaius Octavius (63 B.C.–14 A.D.) in 27 B.C. with the reorganization of the Republic into an Empire.

### Marcomannic Wars (166–180 A.D.)

Event: Following two centuries of stability and economic development after Octavius subverted the Republic into an Empire^4^, numerous Germanic tribes and federations, including the Marcomanni, Quadi, Iazyges, and Suevi lanched attacks across the extent of the northern border^5^. Tribes crossed the Danube and penetrated as far south as Aquileia near the northern coast of the Adriatic Sea. Emperor Marcus Aurelius Antoninus (121–180 A.D.) ultimately repelled the invaders, and his son Lucius Aurelius Commodus (161–192 A.D.) finalized peace arrangements during his reign.

Effects: The war revealed the weaknesses of Rome’s military authority and significantly depleted the treasury. The succession of Aurelius by Commodus marked the transition from peaceful transfer of power to a chaotic sequence of assassinations and instability known as the Crises of the Third Century. Following this period, Gaius Aurelius Valerius Diocletian (244–312 A.D.) reorganized the Empire^6^.

### 3. Gothic Migration (376–410 A.D.)

Event: In 376 A.D., tens of thousands of Gothic peoples begged for permission to cross the Danube south into the Roman Empire in response to the migration of the Huns^7^. The inability to feed all the refugees within the Empire led to a revolt, with the Goths raiding farms and villages. Eastern Emperor Flavius Julius Valens (328–378 A.D.) met them at Adrianople, where the Romans were defeated with Emperor Valens himself falling in battle. Following this the Gothic tribes continue to migrate through the Empire, sacking the city of Rome in 410 A.D. following episodes of violence targeted against Romans of German descent^8^.

Effects: The movement of the Goths permanently destroyed Roman hegemony in the West. Following the sacking of Rome, Germanic tribes carved out kingdoms in Gaul, Thrace, Iberia, and North Africa. The last Roman Emperor, Romulus Agustulus (461–476/507 A.D.), was deposed in 476 A.D. by Odoacer (433–493 A.D.).

### The Migration Period (500–600 A.D.)

Event: Large population movements within Europe introduced Slavic speakers into areas formerly populated by Germans, Romance speakers into areas formerly populated by Thracians and Dacians, and German speakers in areas formerly populated by Romance speakers. Angles and Saxons migrated into Britain, primarily in the south^9^. Additional linguistic groups also migrated, such as the Turcic-speaking Avars, but not all introduced languages persisted.

Effects: The large scale migrations transformed the cultural and linguistic landscape of Late Antiquity, and form the basis of present-day linguistic barriers. This intermixing also resulted in the movement of diseases into Europe^10^. Land use and city occupation became increasingly variable with ephemeral groups^10^. Few primary historical accounts were written during this period, and many earlier accounts were lost. An extended historical overview can be found in the Supplemental Historical Material.

The Roman Empire experienced a decline and revival in the third and fourth centuries A.D., a decisive decline in the first century A.D., and Europe as a whole was the setting for large population movements in the 6th century A.D. While a climatic explanation for these changes has been postulated as early as the 18th century^1^, a clear connection between climate and individual migrations has been lacking. For the Migration Period in particular (c. 500–600 A.D.), there is a clear trend toward cold arid conditions^12, 13^. Palynological data from across Europe indicate an advance of forested lands and a decrease in cereal crops at this time^14–21^. Speleothem data generally indicate more arid conditions^22–24^. However these represent general trends, not necessarily events that would have been recognized as such at the time. Nonetheless, the Migration Period as a social phenomena overlaps with the climatic phenomenon termed the Late Antique Little Ice Age (LALIA)^25^.

A recent reconstruction of the NAO^26^ offers insight into a specific potential climatic driver for historical migrations in Europe. The NAO is the result of the atmospheric pressure difference between the Azores high pressure cell and the Icelandic low. These two pressure cells create a conduit for humid winds which facilitate the development of storms across Europe. The NAO currently drives zonal circulation which can contribute to drought or precipitation in Europe on a regional basis, a role it has likely played since at least the mid-Holocene^27^. A positive North Atlantic Oscillation (NAO+) is associated with wetter conditions in Central Europe and drier conditions in the Mediterranean. In contrast, a negative North Atlantic Oscillation (NAO-) is associated with drier conditions in Central Europe and wetter conditions in the Mediterranean^28, 29^. This forms what has been characterized as a dipole pattern^30^, in which northeastern France, Germany, Scandinavia, northern Poland, and the Baltics undergo a 10th percentile drought. The wind patterns caused by the varying strength of the NAO are a key cause of either drought or surplus precipitation in Europe.

## Results

**Figure 2:**
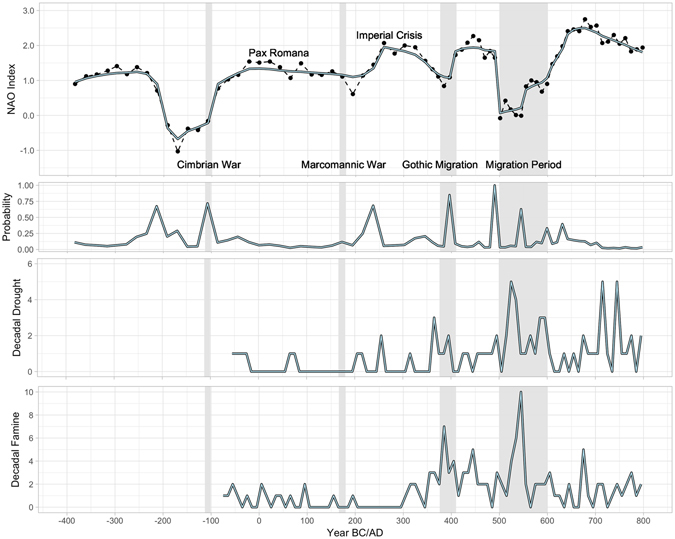
Bayesian change point analysis of NAO^16^ and historical accounts of droughts and famine^23^ with primary migration events. Figure generated in R (3.3.2)^40^.

**Figure 3:**
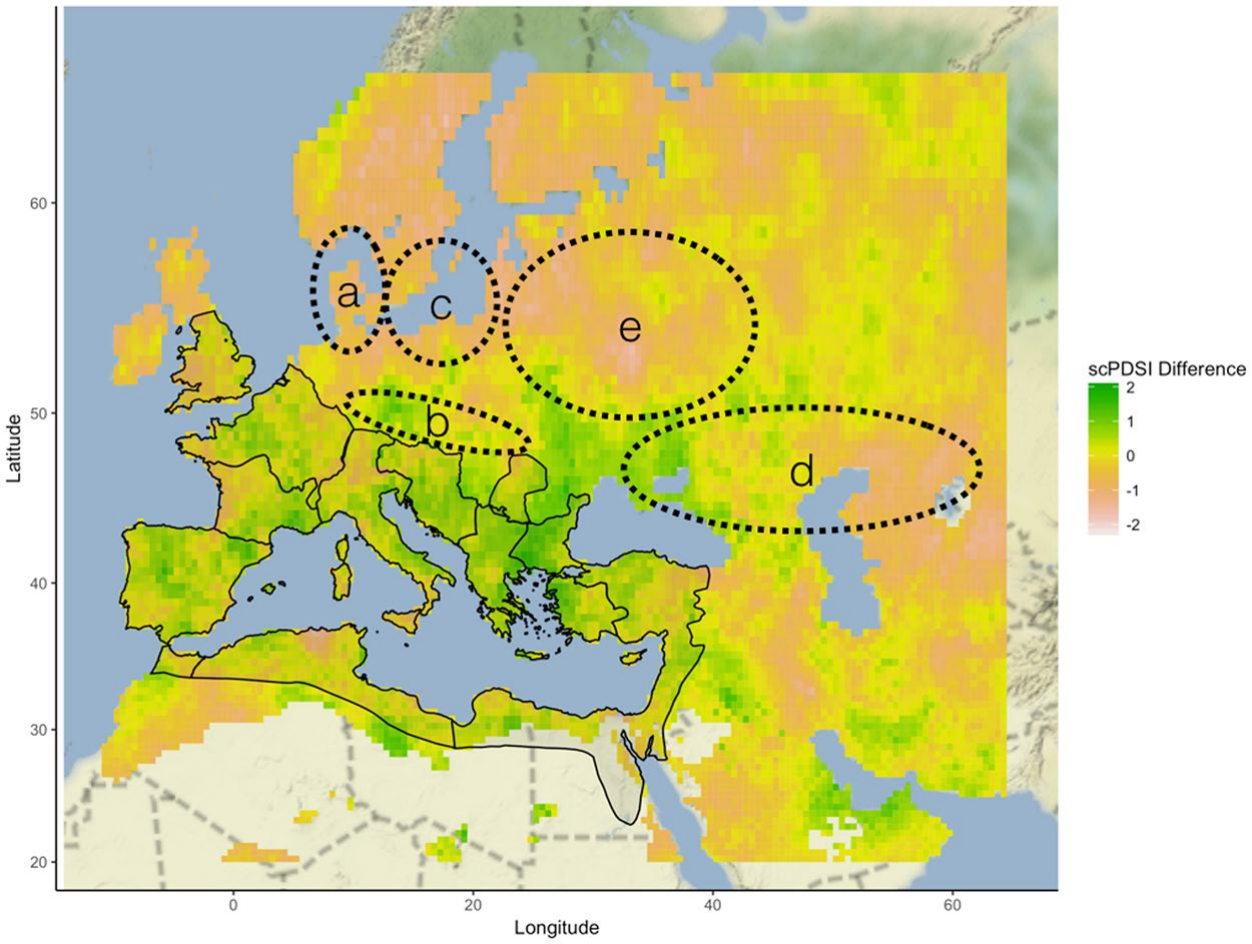
Historical (1900–2014 A.D.) shift in the self-calibrated Palmer Drought Sensitivity Index (scPDSI) from NAO index ranging from 1–2 to an NAO index ranging from 0–1^31, 32^. Areas near the Mediterranean see a shift to wetter conditions, while North Central Europe and the Pontic Steps shift to more arid conditions. Map generated in R (3.3.2)^40^ using map tiles by Stamen Design (under CC BY 3.0. Data by OpenStreetMap, under ODbL) and historic scPDSI records^31, 32^.

There are four NAO shifts which align with historical European migrations in antiquity, with minima at 150 B.C., 190 A.D., 375 A.D., and 500 A.D (Fig. 2). Historically, NAO+ events which ranged from 0–1 are associated with a shift to drought conditions in the territories that primary historical accounts (Tacitus, Strabo, Ammianus Marcellinus, Jordanes) attribute as origins for the tribes which migrated to or past Roman borders (Fig. 3). As drought conditions may have persisted for multiple years or even decades, the incentive to migrate would have been high. Each period in which NAO+ ranges from 0–1 corresponds to one of the four significant Germanic or proto-Germanic migrations (Fig. 2). This suggests that these four historical population migration events may have been a response and strategy to handle inclement agricultural conditions created by a weakened NAO+.

Of these 4 population migration events, the first and last represent the most significant NAO changes by magnitude. The first major historical migration, associated with migrations of the Cimbri and Teutones in 108 B.C., had a Bayesian change point posterior probability of 0.75, though trending toward NAO+ as it fell within the range of 0–1. While NAO- preceded the migrations of the Cimbri and Teutones, these conditions may have been beneficial for their proto-historical homeland in Jutland (Supplemental Fig. 2), while an NAO between 0–1 is historically associate with drought (Fig. 3, Supplemental Figs 1 and 2 ^31, 32^.

By contrast, the Pax Romana (27 B.C.–180 A.D.) had the least variable NAO+, which ranged from +1 to +1.5 (Fig. 2), with the highest Bayesian change point posterior probability associated with the Marcomannic war toward its end. However, Bayesian change point analysis does not indicate this deviation was significant; the NAO reconstruction only has one datapoint registering this change. This period also has few references to droughts or famine in historical accounts (Fig. 2)^23^. Following this tranquil period, the frequency of Nile floods^33^ ﻿and﻿ lake productivity in Lake Holzmaar^34^ declined between 200–300 A.D.^33^. This period is also contemporaneous with the Crises of the Third Century, a period of high turnover in leadership and limited historical records.

**Figure 4:**
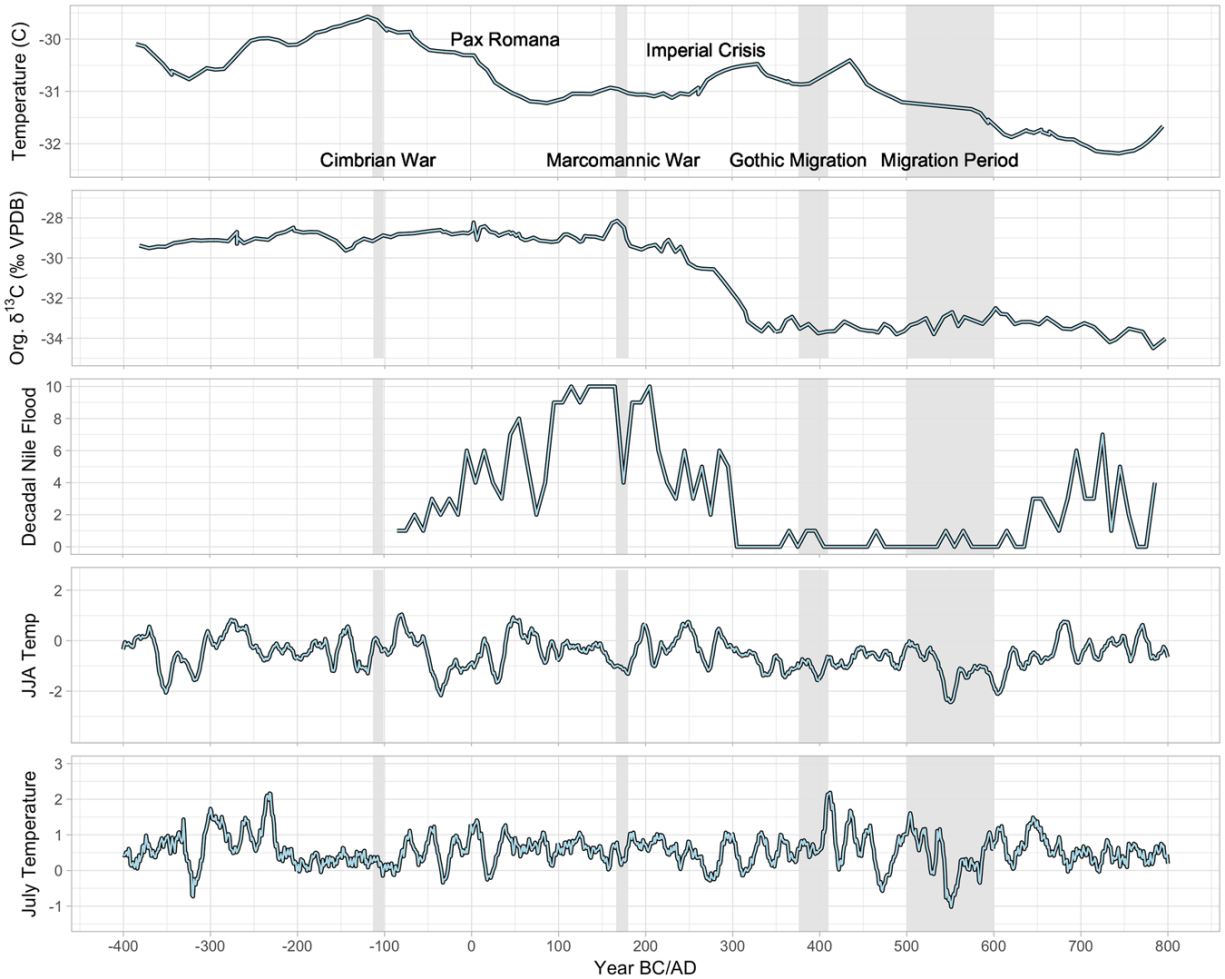
GISP2 Northern Hemisphere Temperature Reconstruction^40^, stable carbon isotope ratios as a proxy for lake productivity from Lake Holzmaar in Germany^34^, summer temperature reconstructions for Northern Europe^12^, July temperature reconstructions for Finland and Scandinavia^13^, and historical Nile flooding events accumulated by McCormick *et al*.^33^ from primary records. Figure generated in R (3.3.2)^40^.

The Gothic Migration south of the Danube in 376 A.D. was associated with a stronger posterior probability of 0.85 toward a weaker NAO+, while the Migration Period had a weakening NAO+ with as high a posterior probability as is possible (0.99) in 490 A.D. A second shift occurred during the Migration Period toward NAO+ at 545 A.D. (0.61). These last two drops in the NAO index, in addition to being associated with large population movements across Europe, are also co-incident with increasing historical accounts of drought and famine (Fig. 2)^33^. Drops in the NAO+ 1–2 range to the NAO+ 0–1 range are associated with a shift to drought conditions for north-central Europe, Scandinavia, and the Pontic Steps (Fig. 3). Historical mentions of drought and famine by historians increase during both the initial Gothic migrations and during the Migration Period (Fig. 2)^33^. The later Migration Period had colder summer temperatures, as reflected by dendroclimatological data from Northern Europe and Scandinavia (Fig. 4)^11^.

Significant migration episodes have high change point probabilities associated with shifts from an NAO+ ranging from 1–2 to an NAO+ ranging from 0–1 (Fig. 2). The largest relative changes would have occurred from summer to winter (Supplemental Figs 3–6). This same change in the instrumental record would predict a shift to drought conditions in territories occupied by the Cimbri, Marcomanni, Goths, Huns, and Slavs in their spatio-temporal context prior to migration (Fig. 3). Shifts to weaker NAO+ conditions may have caused decadal droughts which would have systematically depressed agricultural productivity, creating climatic push factors for migration. The consistently weak NAO+ during the Migration Period was unprecedented for the societies which faced them in both magnitude and duration. While these conditions were associated with drought for tribes living in Northern Europe and the Pontic Steppe, they were more amenable to agriculture within or near Roman borders with a shift to wetter conditions (Fig. 3).

Major migration episodes of entire populations, to be distinguished from smaller individual-based economic migrations^3^, had complex and long-lasting effects on the Roman Republic, then Empire. The first major migration, that of the Cimbri and Teutones in in 108 B.C., precipitated a crises in the Roman Republic^2^. The Republic’s response to this was to reform the military around generals, an arrangement that would have disastrous consequences for the Republic and lead to multiple civil wars and ultimately a collapse of representative government. The Empire which followed enjoyed unprecedented peace during a particularly stable NAO+ period (Fig. 2). The shifts to a weakened NAO+ in 376 A.D. and 500 A.D. led to large population migrations across Eurasia that would not only contribute to the collapse of the Western Roman Empire, but also shift linguistic and cultural boundaries for the following centuries. These differing responses to the same climatic phenomenon over centuries provide a cautionary tale against narrow climatic determinism; the ways in which a society responds to changing conditions affect the outcomes.

A key vulnerability of climate change are push factors which contribute to migrations. The 3-year Syrian drought from 2007–2010 is argued to have influenced agricultural productivity and secondarily food prices, which contributed to the subsequent civil war and refugee crises^35^. Larger food distribution systems are vulnerable to regional drought, as the seperate Chinese drought influenced food prices, which contributed to broader social instability in the Middle East in 2011^36^. For the Arab Spring, drought in food production centers led to increases in food prices which exacerbated existing political tension, causing governments to fall. Ancient accounts from Germanic Tribes indicate that some migrations by Germanic tribes were driven by hunger^37^ (Historical Supplemental Material). Both recent data and the effects of the NAO on the Roman Empire in Late Antiquity suggest that increased risk of regional droughts creates primary and secondary factors for social instability in the short term, and can change social institutions in the long term.

## Methods

The North Atlantic Oscillation proxy record provided by Olsen *et al*.^26^ was assessed relative to the history of migrations into Italy. Bayesian change point analysis was performed using the Barry-Hartigan^38^ algorithm as implemented by Erdman and Edison^39^ in the bcp (4.0) package in the R programming language (3.3.2)^40^. A total of 2000 burn-ins were used with 10,000 Markov-Chain Monte Carlo resimilations of the data to generate posterior means and their associated posterior probabilities for being a change point. All maps and plots in the manuscript and supplemental material were created using the R language; terrain maps were generated from Stamen Design with a creative commons license. Regional boundaries for the Cimbri, Marcomanni and Quadi, Visigoths, Ostrogoths, Huns and Slavs are estimates only and reflect the reliability of the primary historical sources.

Changes in the instrumental climatic records of the 20^th^ and 21^st^ centuries were used to evaluate the potential spatial extent of proxy-reconstructed NAO data. Global gridded self-calibrated Palmer Drought Sensitivity Index (scPDSI) data^31, 32^ were used to analyze spatial patterns of drought following key shifts found in the NAO proxy record^26^. A spatial map of drought of Europe was generated for NAO 1–2 and NAO 0–1 years, with the difference of the two being used to calculate the shift to drought conditions.

Other climatic datasets were included in the analysis, including northern hemisphere temperature reconstructions from the GISP2 ice core^41^, Lake Holzmaar lake productivity^34^, and summer temperature reconstructions derived from tree ring sequences in Central and Northern Europe^12, 13^. For tree-ring data, a running average was taken in 10 year intervals. Historical references to drought, famine, and Nile flooding^23^ were aggregated into 10-year bins to show decadal trends in both.

Datasets and supplemental R code are available for reproducing/extending data analysis.

## Electronic supplementary material

Supplementary Material
