# Supplementary material for: Changes in North Atlantic Oscillation drove Population Migrations and the Collapse of the Western Roman Empire
Source: Sci Rep. 2017 Apr 27;7:1227. doi: 10.1038/s41598-017-01289-z (PMC5430833; doi:10.1038/s41598-017-01289-z)
Supplement: Supplementary file 1 — Supplementary Material [file 41598_2017_1289_MOESM1_ESM.pdf]

Changes in North Atlantic Oscillation drove Population Migrations and the Collapse of the Western Roman Empire

Supplemental Material

Supplemental Historical Background

This brief historical summary of the Roman Republic/Empire is not intended to be a comprehensive overview, but rather provide context for the impacts of shifts in the North Atlantic Oscillation (NAO). Particular attention is focused on how specific migrations/border conflicts affected social developments within the Republic/Empire. It should be noted that border conflicts were common throughout the state's history, however the severity of the risk was variable.

It is impossible to provide a full accounting on how climate has influenced social developments in the Roman Empire, as few climatic changes are documented aside from references to drought and famine in primary historical sources. However, both a historical and archaeological case can be made for significant population movements associated with a weakening of the NAO+ in Europe during four periods: from 113 - 101 B.C., 166 - 180 A.D., 476 - 410 A.D., and 500 - 600 A.D. Weakening of the NAO+ is associated with droughts in modern times; the same climatic drivers may have affected tribes in the same region and encouraged population migration.

Summary

Climate, specifically the varying strength of a positive North Atlantic Oscillation (NAO+), led to climate-based migrations of peoples in north-central Europe and the Baltics. This is based, as discussed in the body of the text, on instrumental observations of the Palmer Drought Sensitivity Index (PDSI) during NAO+ fluctuations of the same magnitude as recorded in the proxy record reported by Olsen and colleagues (2012). Fluctuations in the NAO are linked with the migrations of peoples in these regions (Figure 2) as documented by primarily historical source material:

1. Cimbri and Teutones
2. Marcomanni & Goths
3. Goths & Huns
4. Germanic, Slavic, Romanian, and Turkic speaking groups

Changes in the NAO+ were not necessarily negative within the borders of the Roman Empire - if anything, the Roman Empire benefited from these shifts. It was the Empire's northern neighbors who were exposed to increases in drought conditions. Evidence from historical instrumental records suggests that any change in a weakening of the NAO+ were beneficial within Roman territory but worse outside of it (Figure 3). This would have made Roman territory attractive to those suffering from the effects of drought.

1. The migration of the Cimbri and Teutones between 113 and 101 B.C. presented an existential threat to Rome, to which the consul Gaius Marius responded by forming a permanent military dependent upon generals for compensation. This proved an effective strategy for repulsing the proto-Germanic groups migrating into northern Italy, but proved disastrous for Roman Republic in general and Marius in particular as generals such as Cornelius Sulla used these general-loyal armies to march and seize power on Rome. The fealty of armies to generals fueled civil wars up until Octavius consolidated

power and prevented other generals from enjoying the same unchecked power. In the process, the institutions of republican government were degraded, and an Empire replaced them.

2. The Marcomannic Wars were a broad set of conflicts along the northern Roman borders between 166 - 180 A.D. The Marcomanni were themselves pushed south by movements north by the Goths, as evidenced by the replacement of the Przeworsk culture by that of the Wielbark culture in southern Poland, following the historical outline provided by Jordanes. This movement of peoples pressed south on existing German tribes such as the Quadi, Iazeges, and Marcomanni federation. While this movement would be pushed back successfully by the Romans under Marcus Aurelius, it would come at a tremendous financial cost and years of instability, though there is not a concrete connection between the Marcomannic wars on the frontier and the subsequent destabilization of Imperial control during the third century A.D..
3. A large movement of Germanic tribes under the Visigothic Kings Fritigern and Alavius, fleeing the migrating Huns, pleaded for clemency and sanctuary at the Danube in 376 A.D.. Then Emperor Valens allowed them in, but was unable to provision them, leading to revolt and a significant battle at Adrianople in 378 A.D. where Valens would fall and the Goths would begin migrating within the Roman Empire's boundaries. Subsequent Emperors would be unable to manage the situation, with conditions deteriorating to the point where ethnic violence against Germans would be sanctioned by the Emperor Honorius in 408 A.D. This would prompt the sacking of the city of Rome in 410 A.D., and a permanent collapse of authority in the empire. Subsequent waves of large-scale migration would end the hegemony of the Western Empire.
4. A long period of migration between 500 and 600 A.D., largely outside the scope of primary historical sources, permanently changed the linguistic boundaries of Europe. This would include the movements of Slavic-speaking peoples into the Balkans and central Europe, the integration of Germans with Gallo- and Iberio-Roman peoples, the migration of the Angles and Saxons into Europe, and the (possible) movement of Eastern Roman languages to the coast of the Black Sea and Transylvania. Smaller groups such as the Turkick-speaking Avars and Persian-speaking Alans would also arrive, but not leave a linguistic trace.

As such, the weakening of the NAO+ is consistently associated with significant migrations into southern Europe, from areas associated with drought to those with improving climatic conditions. There are many other significant factors which influence the migration of groups within Europe, but systematic drought conditions lasting a generation or more were strong push factors for population migrations.

Roman Republic (509 - 27 B.C.)

The Roman Republic began sometime before 500 BC, with a likely Etruscan kingdom preceding it. The structure of government within the Republic was a de-facto oligarchy with institutional controls designed to give lower-class citizens a limited say in government through the Tribunate, which was headed first by a Senate and ultimately by two annual Consul appointments. While beginning as a city state, Rome began to overpower its neighbors in the fifth century B.C., a process which would continue until the majority of modern-day Italy fell under its sway in the beginning of the third century B.C. From then on, the majority of Rome's wars would be international, such as the war between the Hellenistic state of Epirus or other Mediterranean powers such as the Carthaginians. There was a ceremonial tradition in Rome to open and close the doors of the Temple of Janus during war and peace, respectively. In 227 B.C., the doors of Janus would be opened and would remain open for 200 years.

The Roman military was uniquely capable of fighting wars over long periods of time, owing to both their adaptability and disciplined formation style. Its military was primarily drawn from landowners who often supplied their own equipment. As Rome conquered its neighbors, it added resources to to its military. The ability to persist through defeat distinguished the Roman military, such as those under both Epirus (282 - 279 B.C.) and later the Carthaginians (264 - 146 B.C.). The most severe of these was the battle of Cannae (216 B.C.), in which 50,000 Roman soldiers were lost. These kinds of defeats were intolerable to other polities, but Rome managed to raise another army and persist in its war, even taking the fight to North

Africa and forcing a retreat from the Carthaginian commander Hannibal despite his victories. This ability to persist despite setbacks set the Roman military apart. The Republic could absorb losses which other states could not.

Following centuries of warfare, the Romans acquired vast wealth from conquered territories. By the second century B.C., it was the unrivaled superpower of the Mediterranean. However, conquered wealth tended to find its way to the patrician class instead of being distributed more broadly. Roman farmers, frequently away from their fields during harvest due to expansionary wars of conquest, found themselves insolvent as wealth poured into the state. This prompted a set of reforms to award soldiers conquered land. Land Reform bills put in place by the Gracchi brothers challenged the relationship between the Tribune and the plebs (1), leading to a division between the *optimates*, or aristocratic classes and the *populares*, or lower classes. Increasing competition for the favors of either side created an environment which allowed populist leaders to rise. Political norms degraded as both *populares* and patrician leaders were killed in mob violence. While the military power of the state remained strong, the social institutions which contributed to Rome's rise were weakening with its expansion. The weakness of Roman norms and traditions was paralleled by the increasing influence of generals on the army.

The growth of complex societies in the Mediterranean region led to a prestige-goods economy at its periphery (2), which increased economic inequality. The nature to this trade served to bind tribal leaders to Rome, while providing in return a way to emphasize their power through luxury items (3). This policy created a set of small buffer states which could serve the defenses of the growing Roman power (4). While not a part of the Roman political state per se, these regions were strongly shaped by Roman influence and material culture.

Cimbrian War (113 - 101 B.C.)

The Cimbri people's originated in north-central Europe, not far from the modern-day Denmark (Figure 1) (5). Marching south, they joined forces with the Teutones and continued to the outer border of the Roman Republic. The arrival of these proto-Germanic tribes was a shock to the Romans, who were previously unaware of their presence but now faced an existential threat to the state. They first invaded the allied Taurisci, who called for Roman legions for defense. Rome sent an army army led by consul Gnaeus Papirius Carbo (? - 112/111 B.C.), which met the Cimbri and Teutones at Noricum in 112 B.C. Carbo initially succeeded in ordering the proto-Germanic tribes to vacate the area, but decided to ambush them despite their compliance with the demand. In the following battle, the Roman Legions were defeated, and the tribes continued into Gaul. While invasion was not imminent, Rome's vulnerability to mass migration was revealed. The tribes next encountered Roman armies in 109 B.C. at Gallia Narbonensis in present-day southern France and Bordigala (present-day Bordeaux) and defeated them in both battles.

The collapsing defenses at the northern borders of Rome caused considerable panic, of which Gaius Marius (157 - 86 B.C.) was the beneficiary. Marius, while born to a humble farming family, rose through the ranks of the legions and had an opportunity to serve along side Publius Cornellius Scipio Amellianus Africanus (185 - 129 B.C.), who selected the young legionary for leadership. Marius was active (though not always successful) in politics as a *populare*, The Jugurthine War in North Africa (112 - 106 B.C.) led to a disagreement between Marius and his commander Quintus Caecilius Metellus (160 - 91 B.C.). Marius' popularity with the troops and promises of victory in the war led to his first election as consul in 108 B.C.

Marius, faced with a war in North Africa and a large population migration just north of Italy, began an ambitious program to reform the Roman army. At the time, land-ownership was a prerequisite of service in the military. This had been the source of frustration which early *populare* reformers had tried to address; during the expansion of Rome's territories the patrician class (or *optimates*) had sent farmers far from their lands for years at a time to conquer foreign holdings while retaining the majority of the material rewards from their victories. As farms went bankrupt and the *optimates* grew rich, the Roman

military became increasingly unable to cope with the demands of a far-flung empire. To address this, Marius opened up service to all willing Roman citizens without regard for their financial means to procure their own arms. This swelled the ranks of the legions, enabling the Romans to more effectively respond to military threats. The growing army would be armed by the state for 16 year enlistments, which would in effect create a mass employment system for the poor. This enabled a standing army, which could immediately respond to threats as they emerged. Full compensation for the army would come in the form of land grants and pensions from their general. These changes would solve a host of military and economic problems for the Roman Republic, enabling it to quickly respond to existential threats like that provided by the Cimbri and Teutones. However, it shifted the loyalty of thousands of citizens of Rome from the state to their particular commanding general, who would be responsible for their financial well being and, in case of victory, enrichment. As Plutarch (6) recounts:

> "Contrary to law and custom he enlisted many a poor and insignificant man, although former commanders had not accepted such persons, but bestowed arms, just as they would any other honour, only on those whose property assessment made them worthy to receive these, each soldier being supposed to put his substance in pledge to the state" (Plutarch IX: 483)

Marius directed his attention to the war in North Africa, who he would eventually defeat in 105 B.C. with the help of his quaestor Lucius Cornelius Sulla Felix (138 - 78 B.C.) who would capture Jugurtha. During this time, the Cimbri and Teutones would continue their migrations, and in 106 B.C. would draw out the Roman Legions once again. The two commanders, Gnaeus Mallius Maximus and Quintus Servilius Caepo, were deeply at odds with the former being a *populare* and the second being an *optimate*, who felt he should not have to serve someone born a commoner. In 105 B.C., at Arausio in present-day southeastern France, the respective armies under each commander camped separately. Caepo pressed an attack against the proto-Germanic tribes during their negotiations with Maximus; the attack ended in defeat for Caepo. With one half the army eliminated, the Cimbri turned on the army of Maximus while it was positioned against the Rhône river. The result of the battle was the loss of close to 80,000 Roman troops and a collapse of the northern defenses of Italy.

Marius, fresh off his victory against Jugurtha, was elected consul for the second time during the crises while absent of Rome and in violation of an earlier law preventing two consulships within a decade of each other. The proto-Germanic tribes, however, divided and move to new territory in the Iberian peninsula, providing time for the Romans to recover. Marius was elected to still another consulship in 103 and 102 B.C., when the proto-Germanic tribes proceeded towards Italy. They divided their forces while crossing the Alps, allowing Marius to advance against each group separately. He met the Teutones in Narbonensis, where the tribes attacked separately. Marius held against a first strike by the Ambrones while laying an ultimately successful ambush for the remainder of the Teutones forces. The loss to the Teutones was reportedly total, with over 100,000 killed.

At present-day Brenner Pass between Italy and Austria, the Cimbri defeated a Roman army and continued into Northern Italy. Marius, elected to yet another consulship in 101 B.C., marched north to meet them at Vercellae. There Marius defeated the last remaining threat to Rome, with 65,000 dead. The Roman histories reported the women of the Cimbri killing each other and their children in a mass suicide to avoid slavery at the hands of their enemies. This particular note in the histories highlight that while this event was a military invasion to the Romans, it was a population migration for the Germanic tribes. As Plutarch (6) recounts:

> "The fugitives, however, were driven back to their entrenchments, where the Romans beheld a most tragic spectacle. The women, in black garments, stood at the waggons and slew the fugitives — their husbands or brothers or fathers, then strangled their little children and cast them beneath the wheels of the waggons or the feet of the cattle, and then cut their own throats. It is said that one woman hung dangling from the tip of a waggon-pole, with her children tied to either ankle; while the men, for lack of trees, fastened themselves by the neck to the horns of the cattle, or to

> their legs, then plied the goad, and were dragged or trampled to death as the cattle dashed away." (Plutarch XXVII: 537)

Roman Revolution (101 B.C. - 27 B.C.)

Following the Cimbri defeat at Vercellae, Marius granted allied Italian soldiers Roman citizenship without first consulting the Senate. The resulting shift in status would polarize people in Rome, and lead to increased demands by allied Italian city-states for increased rights under the Republic. Tensions would build for the following years until a decree demanding the expulsion of non-citizens from Rome passed in 95 B.C. As Latin allies to Rome considered their options, a politician, Marcus Livius Druses, was assassinated in 91 B.C. following a proposal to offer citizenship to allied city states; this led directly to war. Multiple allied city-states declared war on Rome. Rome first attempted to hold territory while trying to retain allies who had not yet joined the revolt. The war would claim hundreds of thousands of lives in Italy, Caepo's among them. Cornelius Sulla, Marius' former quaestor, played an instrumental role in maintaining the fragile Roman alliance system through victory against the revolting Latin allies. Ironically, the Latin states which remained with Rome would be granted citizenship for their assistance in the struggle.

As the Social Wars came to a conclusion, war broke out in Anatolia as Mithridates VI of Pontus (134 - 63 B.C.) invaded Greece. Sulla received a consulship in 88 B.C., and with it the expectation he would lead the war against Mithridates. Marius desired this command however, and compelled the Tribune to appoint him instead. Sulla ignored this and proceeded against Mithridates, successfully convincing his army to ignore the dictates of the Roman government. Then, instead of marching East, Sulla marched on Rome. Marius narrowly escaped with his life as Sulla passed a death sentence against his former commander. Sulla left Rome to fight Mithridates as allies of Marius received the consulships of 87 B.C., including Lucius Cornelius Cinna (? - 84 B.C.). He would serve the consulship in the next year as well alongside Marius in his seventh. Marius and Cinna would drive the murders of loyalists to Sulla, real or suspected. Marius would himself die of natural causes days into his term (6).

Sulla returned to Rome in 83 B.C. in a second march against the city, still held by Cinna. The city sent troops to stop him, but they were no match for Sulla's veterans. Romans siding with Sulla left the city to contribute to his army, including Marcus Licinius Crassus (115 - 53 B.C.) and a young Gnaius Pompeius (106 - 58 B.C.). After multiple skirmishes, Sulla would win this brief civil war at the Colline Gate of Rome, with 50,000 combatants dead. Sulla would then be appointed dictator by the Senate, and would proceed with a bloody proscription against his enemies, which would later devolve into a general period of assassination by various individuals looking to respond to grievances or simply seize property. One notable person would be spared from the proscriptions, Cinna's son-in-law Gaius Julius Caesar (100 - 44 B.C.), despite being a *populare* in a time of overwhelming victory for the *optimates*. Following his disruptive reign as dictator, Sulla retired from the dictatorship in 81 B.C. and stood for the consulship in 80 B.C., and following one term, retired again. While there was an apparent return to normal politics in Rome, the events beginning with the Cimbri invasion and Marian reforms would permanently damage Roman institutions - it was now apparent to all aspiring politicians that a loyal army was critical to obtaining power.

Crassus, Pompeius, and Caesar would heed these lessons years later as civil wars resumed. They would form an uneasy triumvirate alliance with each other from 60 B.C. to 53 B.C. to pursue their respective ambitions. Each would attempt to conquer new territory to reward their troops - Crassus in Mesopotamia, Pompeius in the Levant, and Caeser in Gaul. Crassus would die in a botched invasion of Parthia while Pompeius enjoyed the riches of Palestine and Caesar pulled Gaul and Britain into the Roman orbit. Civil war would erupt once again in 50 B.C. when the Senate under Pompeius demanded that Caesar disband his army before returning to Rome. Caesar instead rapidly pushed for Rome, forcing Pompeius and the Senate to flee. The war would last until 45 B.C., with Caesar defeating the Senate armies and having himself declared dictator. His assassination in 44 B.C. and surprise selection of a young Gaius Octavius

(63 B.C. - 14 A.D.) as his heir over his trusted ally in the Gallic and Civil Wars Marcus Antonius (83 - 30 B.C.). This led to a brief war between them, with Octavius defeating Antonius the following year. The Senate would attempt to restore things to order following this brief outburst of conflict, resulting in a surprising second triumvirate in 43 B.C. between Octavius, Antonius, and Marcus Aemilius Lepidus (88/89 - 12 B.C.) to retain power and revenge Caesar's assassination. They returned to the proscriptions used by Sulla a generation earlier to pursue a policy of murder and seizure of assets. These funds would be used to fight a war against the Senate loyalists and Caesar assassins Marcus Junius Brutus (85 - 42 B.C.) and Gaius Cassius Longinus (85 - 42 B.C.), which would conclude in 42 B.C. with the victory of the Caesarians (6).

The uneasy alliance between the second triumvirate did not break into war immediately as Octavius fought against a pirate empire based in Sicily under the leadership of Pompeius' son, Sextus Pompeius (67 - 35 B.C.), who had been radicalized following his father's murder. Antonius fought a brief war against Parthia to the east. Tensions would rise in 33 B.C., with Octavius portraying himself as the defender of traditional Roman mores while Antony presented himself as a pharaoh in Egypt with Caesar's former lover Cleopatra VII Philapator (69 - 30 B.C.). War broke out in 31 B.C. Octavius was quickly victorious in conquering cities loyal to Antonius in Greece and that same year won a decisive naval victory at Actium. He invaded Egypt in 30 B.C. and met little resistance.

Octavius would, on the face of it, return control to the Senate in 27 B.C. following the last civil war. In exchange, the Senate supported Octavius' control over most of Roman provinces, save for Africa, Illyria, and the Balkans. Octavius was renamed Augustus and also granted the status of *principes*, to emphasize his status as simply a citizen and avoid connotations with monarchy. This word would be the origin of the English word prince. He would ceremonially close the doors of the Temple Janus in 27 B.C. At all steps, Octavius was cautious to avoid appearing a monarch, as he recounts himself in the Res Gestae (7):

> "The dictatorship offered me by the people and the Roman Senate, in my absence and later when present, in the consulship of Marcus Marcellus and Lucius Arruntius I did not accept. I did not decline at a time of the greatest scarcity of grain the charge of the grain-supply, which I so administered that, within a few days, I freed the entire people, at my own expense, from the fear and danger in which they were. The consulship, either yearly or for life, then offered me I did not accept." (Augustus 5: 353)

Pax Romana (27 B.C. - 180 A.D.)

The early Roman Empire was not a formalized as such at its conception. Power was not necessarily absolute and centered in Octavius, but rather emerged from the merging of earlier positions such as Tribune, Censor, and Pontifex Maximus. The Senate did not end, nor would it until well after the collapse of the Western Empire. The initial transition to Romans at the time would have been from a century of civil war to stability. The Empire would be established under Octavius due in part to his long life, this would also undercut the stability of succession as he outlived many of his likely heirs. While an adopted son of Octavius, Tiberius Clauidius Nero (47 B.C. - 37 A.D.) would succeed him, later Emperors would rely on Senate approval for ascension. The earlier political distinction between the earlier *optimates* and *populares* would be replaced by the social class distinctions between the *honestiores*, or aristocratic class, and the lower class *humilores*.

The Julian dynasty, starting with Augustus, would not last long after his death, giving way to competition between generals after 69 A.D. A short lived Flavian dynasty would follow, consisting of former generals Vespasian (9 - 79 A.D.) and his sons Titus (39 - 81 A.D.) and Domitian (24 - 96 A.D.). The following Antonine dynasty was widely regarded as the peak of the Empire. It reached its largest territorial extent under Marcus Ulpius Traianus (53 - 117 A.D) in 98 - 117 A.D (Figure 1).

Over the decades, the Roman Empire would lead to increased social mobility when measured over generations, as evidenced by the collapsing fortunes of former patrician families and the election of emperors such as Traianus with no patrician ancestry. This period would also feature migration within the empire (8), likely as a consequence of economic opportunities.

At the periphery of the Empire were Germanic peoples, for whom contact increased with the expansion of Rome into Gaul. A failed attempt at expanding into Germania by Augustus resulted in a battlefield loss in 9 A.D. and a more or less permanent northern border for the Roman Empire. Publius Cornellius Tacitus (56 - 120 A.D.) describes German Tribes at his time (9):

> "It is well known that none of the German tribes live in cities, that even individually they do not permit houses to touch each other: they live separated and scattered, accordingly as spring-water, meadow, or grove appeals to each man: they lay out their villages not after our fashion, with buildings contiguous and connected; everyone keeps a clear space around his house, whether it be a precaution against the chances of fire, or just ignorance of building." (Tacitus Germania: 287)

Tacitus also suggested their agricultural system was less complex than that of the Romans, arguing that

> "Land is taken up by the village as a whole, in quality according to the number of the cultivators; they then distribute it among themselves on the basis of rank, such distribution being made easy by the extent of domain occupied. They change the arable land yearly, and there is still land to spare, for they do not strain the fertility and resources of the soil by tasking them, through the planting of vinyards, the setting apart of water-meadows, the irrigation of vegetable gardens. Grain is the only harvest required of the land; accordingly the year itself is not divided into as many parts as us; winter, spring, summer, have a meaning and a name; of autumn, the name alike and boundaries are unknown." (Tacitus Germania: 301)

While difficult to evaluate the veracity or generalizability of Tacitus' claim, this indicates that annual climatic factors, rather than seasonal, would have the biggest impact on the subsistence of German tribes at this time. Earlier comments by Caesar reflect a simpler agricultural system, at least compared to the Celts (10):

> "They are not devoted to agriculture, and the greater portion of their food consists of milk, cheese, and flesh. No one owns a particular piece of land, with fixed limits, but each year the magistrates and the chiefs assign to the clans and the bands of kinsmen who have assembled together as much land as they think proper, and in whatever place they desire, and the next year compel them to move to some other place. They give many reasons for this custom---that the people may not lose their zeal for war through habits established by prolonged attention to the cultivation of the soil; that they may not be eager to acquire large possessions, and that the stronger may not drive the weaker from their property; that they may not build too carefully, in order to avoid cold and heat; that the love of money may not spring up, from which arise quarrels and dissensions; and, finally, that the common people may live in contentment, since each person sees that his wealth is kept equal to that of the most powerful." (Caesar VI.22: 347)

Funerary rights described by Tacitus suggest minimal grave goods were used and that remains were cremated, which is broadly consistent with contemporary descriptions of the Przeworsk cultural complex in present-day southern Poland (11). Tacitus identifies several groups such as the Chauci, Catti, Suevi, Langobardi, Marcomanni, Quadi, and Gothones. He also identifies a remaining population of the Cimbri as a German tribe.

Marcomannic Wars (161 - 180 A.D.)

The peace and stability of the Pax Romana began to waiver in 161-166 A.D., with the Empire was at war on multiple fronts and suffered millions of casualties due to the Antonine plague during the reign of Marcus Aurelius (121 - 180 A.D.). The northern border was attacked by multiple tribes, including the Chatti and Chauci. A ceasefire was arranged by the leader of a confederation of tribes called the Marcomanni (Figure X1b). The Marcomanni appear to have been a nomadic group which, at the time of conflict, found themselves pressed against the Roman border in present-day Czech Republic and southern Poland. The Roman geographer Strabo explicitly connects their wandering to that of the earlier Cimbri (5):

> "All these nations easily change their abode, on account of the scantiness of provisions, and because they neither cultivate the lands nor accumulate wealth, but dwell in miserable huts, and satisfy their wants from day to day, the most part of their food being supplied by the herd, as amongst the nomade races, and in imitation of them they transfer their households in waggons, wandering with their cattle to any place which may appear most advantageous. There are many other smaller German tribes, as the Cherusci, Chatti, Gamabrivi, Chattuarii, and next the ocean the Sicambri, Chaubi, Bructeri, Cimbri, Cauci, Caulci, Campsiani, and many others." (Strabo VII. 1: 445)

The Gothic historian Jordanes (12) suggested that at this time the Goths were expanding in the Baltics following their departure from Scandinavia. Contemporaneously, there was a rapid expansion of Wielbark culture in the first and second centuries into southern Poland. The earlier archaeological complex, that of the Przeworsk culture, is typified by cremation of both remains and burial goods, though there are occasional warrior burials (13). The Wielbark culture, by contrast, is defined by more frequent inhumations and more complex funerary architecture such as stone circles and pavements (14). This archaeological change has been connected to the movement of the Goths (15), though the delineation between historical group, ethnic affiliations, and archaeology should not be casually conflated. Nonetheless, the movements of material culture identified by archaeological investigations matches the historical description by Jordanes (12) of a movement of Goths from Scandinavia to the Baltic coast of present-day Poland, and with the migration of other Germanic groups further south.

In 169 A.D., conflict began again as the Iazyges defeated the Romans on the Dacian border while Costoboci invaded Thrace. To the west, the Marcomanni invaded under the leadership of Ballomar and defeated an army of 20,000 Romans at Carnuntum near the Danube. They then attacked the town of Aquileia, defeating Roman forces sent to defend it. Emperor Marcus Aurelius sent forces to retake Aquileia while he made peace with various other invading tribes. Over the following years those tribes, namely the Quadi and Iazyges, would re-open hostilities, but the Roman Empire could defeat them individually. In 177 A.D., the Quadi and Marcomanni both revolted, and were defeated completely in 180 A.D.. It was the migrations of the Germanic tribes at the borders of Rome, not just armies of male soldiers, which drove conflict between those with defined territory (Romans) and those without (Iazyges et al.). As Cassius Dio (16) noted:

> "Many of the Germans, too, from across the Rhine, advanced as far as Italy and inflicted many injuries upon the Romans. They were in turn attacked by Marcus, who opposed to them his lieutenants Pompeianus and Pertinax ; and Pertinax (who later became emperor) greatly distinguished himself. Among the corpses of the barbarians there were found even women's bodies in armor." (Cassius Dio 72: 12-13)

Macus Aurelius did not have time to enjoy his victory, he died that same year in 180 A.D. His son and heir, Lucius Aelius Aurelius Commodus (161 - 192 A.D.) would quickly conclude a peace with the Quadi and Iazyges and returned to Rome to celebrate a triumph to mark the occasion. The movement of the Marcomanni, Quadi, and Iazyges was explicitly connected to the much earlier movements of the Cimbri and Teutones as unique threats to Rome, as recorded by Ammianus Marcelius (17):

> “The Teutones and the Cimbri came suddenly from the remote shores of the ocean, and overran Italy; but, after having inflicted enormous disasters on the Roman republic, they were at last overcome by our illustrious generals, and being wholly vanquished, learnt by their ultimate destruction what martial valour, combined with skill, can effect. Again, in the reign of the Emperor Marcus Aurelius, the insane fury of a number of different nations combined together, after fearful wars ... would have left but a small part of them.” (Ammianus Marcellus XXXI.V.12: 591)

The Roman Empire had survived the earlier two, though the threat of the Cimbri and Teutones had caused the institutions of the Roman Republic’s army to buckle and break, shaping the remainder of that government’s duration. There is no direct connection between the Marcomannic wars and the sustained crises in government which followed, but the victory came at great expense to the Empire not only militarily, but also financially, as Eutropius (18) recounts:

> “Having persevered, therefore, with the greatest labour and patience, for three whole years at Carnuntum, he brought the Marcomannic war to an end; a war which the Quadi, Vandals, Sarmatians, Suevi, and all the barbarians in that quarter, had joined with the Marcomanui in raising; he killed several thousand men, and, having delivered the Pannonians from slavery, triumphed a second time at Rome with his son Commodus Antoninus, whom he had previously made Caesar. As he had no money to give his soldiers, in consequence of the treasury having been exhausted for the support of the war, and as he was unwilling to lay any tax on the provinces or the senate, he sold off all his imperial furniture and decorations, by an auction held in the forum of the emperor Trajan, consisting of vessels of gold, cups of crystal and murrha, silk garments belonging to his wife and himself, embroidered with gold, and numbers of jewelled ornaments. This sale was continued through two successive months, and a great quantity of money was raised from it. After his victory, however, he gave back the money to such of the purchasers as were willing to restore what they had bought.” (Eutropius VIII.13: 182 - 183)

Imperial Crisis (192 - 284 A.D.)

Marcus Aurelius was characterized as the last of the so-called good emperors by later historians. After his death the Empire grew increasingly unstable, beginning with the rule of Commodus between 180 and 192 A.D., a period, according to historian Cassius Dio (16), in which Rome’s history “descends from a kingdom of gold to one of iron and rust”. Commodus’ reign was accompanied by increasing internal strife, and by the end of his reign he had proclaimed himself the son of Jupiter.

The assassination of Commodus in 192 A.D. would do little to quell the situation, instead it led directly to a series of succession crises. Five emperors would serve within a year. The successor to this instability, Lucius Septimus Severus (145 - 211), was assassinated in 211 A.D., setting of a crises that would last half a century; 26 men would serve as emperor during this time. Perhaps the severest political calamity was the capture of Emperor Publius Licinius Valerianus (193/195/200 - 260/264 A.D.) in 260 A.D. by the Sassanian Persians. The Empire split into three political entities following this event; a Palmyrene Empire to the East, a Gallic Empire to the west, and the remains of the Roman Empire in the middle suffering from barbarian incursions to the north. The ascension of Lucius Domitius Aurelianus (214/215 - 275 A.D.) in 270 A.D. marked a turning point in the Empire’s fortunes; his wars in the East and West successively restored the Empire’s former’s borders while ejecting invasion forces. Aurelian was murdered in 275 A.D., sending the Empire back into crises.

The Empire was re-organized by Gaius Aurelius Valerius Diocletianus (244 - 312 A.D.) in 293 A.D. into a form of rule known as the Tetrarchy (19). The Empire was divided in two, with each Emperor having a co-emperor. This system degraded into civil war, with Flavius Valerius Aurelius Constantinus (272 - 337 A.D.) winning in 306 A.D. This would mark the beginning of Christianity as a political force within the

Empire. Constantine moved the capital to Byzantium (Constantinople/Istanbul) in 330 A.D. This period marked the split between the Eastern and Western Roman Empires.

Gothic Migration (376 - 410 A.D.)

In 376 A.D., tens of thousands of Germanic and Persian speaking peoples, collectively referred to by later historians as the Visigoths, showed up on the borders of the Roman Empire asking for shelter. The origins of these tribes lay in north-central Europe and Scandinavia (Figure 1) Their leaders, Fritigern (? - 382 A.D.) and Alavivius (? - 376 A.D.) were fleeing a group of people they called the Huns. Ammianus Marcellinus (17) recounts:

> "While this important work was going on, the Huns kept pressing on his traces with great speed, and they would have overtaken and destroyed him if they had not been forced to abandon the pursuit from being impeded by the great quantity of their booty. In the mean time a report spread extensively through the other nations of the Goths, that a race of men, hitherto unknown, had suddenly descended like a whirlwind from the lofty mountains, as if they had risen from some secret recess of the earth, and were ravaging and destroying everything which came in their way. And then the greater part of the population which, because of their want of necessaries had deserted Athanaric, resolved to flee and to seek a home remote from all knowledge of the barbarians; and after a long deliberation where to fix their abode, they resolved that a retreat into Thrace was the most suitable for these two reasons: first of all, because it is a district most fertile in grass; and also because, by the great breadth of the Danube, it is wholly separated from the barbarians, who were already exposed to the thunderbolts of foreign warfare. And the whole population of the tribe adopted this resolution unanimously." (Ammianus Marcellus XXXI.III.8: 591)

Emperor Flavius Julius Valens (328 - 378 A.D.) of the Eastern Empire permitted their passing, hoping they could defend the northern border against this new foe. Unfortunately, entering Roman lands did little to improve the situation of the Goths. They were placed into large and poorly provisioned camps. As recounted by Jordanes (12):

> "Soon famine and want came upon them, as often happens to a people not yet well settled in a country. Their princes and the leaders who ruled them in place of kings, that is Fritigern, Alatheus and Safrac, began to lament the plight of their army and begged Lupicinus and Maximus, the Roman commanders, to open a market." (Jordanes XXVI: 134-135)

However, relations quickly deteriorated between the Roman commanders and the Visigoths likely due to mutual suspicion and limited food. The Visigoths began raiding farms and settlements in Thrace, in the present-day Balkans. The Western Emperor Flavius Gratianus (359 - 383 A.D.) sent forces to help contain the large tribes now loose within the Empire's borders. In 378 A.D. Emperor Valens met the Visigoths at Adrianople, where he and the Eastern Roman Army was crushed. Emperor Valens himself would be a casualty of this battle. From this point on, the northern defenses of the Roman Empire were permanently broken. While the Visigoths could not take Adrianople itself, they were able to carve out portions of the Empire for themselves. Other Germanic tribes would hear of their success. While the defeat was the Eastern Empire's, the consequences were severe for the Western Empire. Successive Roman Emperors would have to negotiate with Gothic tribes as partners in the Empire.

Evidence for an increased presence of Goths south of the Danube is evidenced by double-plated brooches consistent with the Chernyakhov material culture complex (19). The Chernyakhov culture, while distinct from the Wielbark culture, shares strong similarities for burial styles, with an arguable direct overlaps (21). Dental analysis of burial remains finds no difference between members of the two cultures (21). This cultural complex south of the Danube split the Eastern and Western Empires, in addition to opening a route of migration to the West for Germanic and Hunnic tribes.

The Visigoths under Alaric I (370/375 - 410 A.D.) would begin a sequence of attacks against the Roman Empire, invading Thrace in 395 A.D. They were stopped by Flavius Stilicho (359 - 408 A.D.), a half-Vandal who had, earlier in life, served alongside Alaric when he was an auxiliary troop within the military. Stilicho, now the highest ranking official in Roman History of German descent, was serving as regent for the young Emperor Honorius (384 - 423 A.D.). Stilicho would repel Alaric from Thrace that year, and again in Macedonia in 397 A.D. Stilicho would be appointed consul in 400 A.D. Alaric would invade Italy in 401 A.D., and suffer another defeat by Stilicho that same year. For his services, Silicho would be awarded a Triumph, both the first person of German descent and last person to ever receive this award.

Alaric first led the Goths into Greece, where they began attacking cities such as Athens and Thebes. The Empire was unable to mount a response to these provocations in Greece, as large group of Germans and Celts were preparing to cross the Danube into the Empire. The Roman general and former regent Stilicho was sent to cross the Danube before them and, in a surprise attack, disperse their forces.

Multiple German tribes, including the Alans, Suevi, and Vandals, would be pushed back by Stilicho in 405 A.D. However, in this war Stilicho had drawn heavily from defenses in Gaul, for which those same tribes would enter Rome in 406 A.D. while the Rhine was frozen. Stilicho would be unable to effectively respond, and instead choose to defend Italy in the Alps. In Ravenna, his inability to handle the collapse of Gail and the threat of war with Alaric led to an army revolt which would claim his life and his son. Social prejudices against Germans may have also played a role, as after his death Honorius urged Romans to attack Germans throughout the Empire, as Zosimus (23) reports:

> "The soldiers who were in the city, on hearing of the death of Stilico, fell upon all the women and children in the city, who belonged to the Barbarians. Having, as by a preconcerted signal, destroyed every individual of them, they plundered them of all they possessed. When this was known to the relations of those who were murdered, they assembled together from all quarters. Being highly incensed against the Romans for so impious a breach of the promises they had made in the presence of the gods, they all resolved to join with Alaric, and to assist him in a war against Rome." (Zosimus V: 161).

These actions incensed the Goths, who pushed for a renewed attack on Italy. Alaric faced no serious opposition in his march to Italy with the legions in shambles post-Stilicho, and began the siege of Rome that year. Alaric demanded the freedom of 40,000 Gothic slaves and command of the Roman armies. The Senate freed the slaves, but Honorious did not give Alaric command of the army. The siege was renewed in 409 A.D., and following a failure in a second set of negations, Alaric proceeded to sack Rome in 410 A.D. The Goths would continue as far south as Sicily, significantly weakening the Western Roman Empire. The city of Rome had not been violated since the Celts attacked in the fourth century B.C. (excluding Sulla and Caesar). The Gothic historian Jordanes (12) noted that they took care not to burn the city. From this point on, the Roman Empire was fatally fractured in the West. Rome would again be unable to defend itself 4 years later when the new Gothic king Athavulf (370 - 415 A.D.) sacked it a second time before continuing to split Gaul from the Empire. Leadership of the Goths would be chaotic in the following years with high overturn, but Rome would no longer be the dominant military force within its own borders

Collapse of the Empire (410 - 493 A.D.)

Following the Gothic sack of Rome, the surviving government was unable to administer all provinces. Britain was more or less abandoned, with few historical records accounting for changes which took place on the island afterwards. The Iberian peninsula was invaded by the Suebi (descendants of the Marcomanni and Quadi), Vandals, and Alans in 409 A.D. The Roman Empire in 418 A.D. offered the Visigoths land in Gaul to help buffer the threat, seeding a Visigothic kingdom along the Atlantic coast.

This was, however, more a recognition of the Visigothic presence in Gaul than anything else. Visigoths would over the course of the following century expand into Iberia and push the Vandals and Alans into North Africa.

The weakness of the northern borders led other groups to invade the Western Empire, the Anglo-Saxons in England, the Franks in Gaul, and the Alemanni in Germania. The Huns, who had initially driven the Visigoths to the Roman border in 376 A.D., had begun to attack the Eastern Empire as early as 395 A.D., with some Huns serving in the Roman Legions. However, they were small, isolated, but formidable, bands which controlled a wide swath of territory in the central European basin. They would unite under the brothers Bleda and Atilla (406 - 453 A.D.) in 434 A.D., with Atilla taking full control in 445 A.D. In 450 A.D., Honorius, sister to Western Roman Emperor Flavius Placidius Valentianianus III (419 - 455 A.D.), sent an invitation to marriage to Atilla. This prompted Atilla to lay claim to half the Western Roman Empire, and he invaded with client groups of Franks, Goths, and Burgundians. Atilla would be turned back by a Visigoth force near Troyes in modern-day France led by Theoderic I (390/393 - 451 A.D.), who would die in the fighting. This would only be a temporary setback, as the following year Atilla attacked Aquiela, permanently destroying the city's prominence in the Western Roman Empire. Atilla would make it to Rome, but would be turned away by Pope Leo I who successfully negotiated a peace with no concessions, though Rome at the time was experiencing a famine and could not support the number of foreign troops (24).

The Hunnic kingdom would not last after Atilla's death in 453 A.D. The Gepids would rise against the Huns and in 454 reject their control alongside other Gothic tribes, ending Hun supremacy in Eastern Europe. However, one Hunnish-allied group, the Salian Franks, would conquer a small portion of Roman Gaul under Childeric I (440 - 481/482 A.D.) and establish the founding French Merovingian dynasty.

The last Roman Emperor, Romulus Augustulus (461 - 476/507 A.D.), would be deposed by Flavius Odoacer (433 - 493 A.D.) in 476 A.D., who founded the Kingdom of Italy with the approval of the Roman Senate. This kingdom would be conquered by Theoderic (454 - 524 A.D.) of the Ostrogoths in 493 A.D., cementing Germanic control over the remnants of the Western Roman Empire. Famine would continue to play a role in the movements of the Goths in terminal antiquity, as reported by Jordanes (12):

> "Then as the spoil taken from one and another of the neighboring tribes diminished, the Goths began to lack food and clothing, and peace became distasteful to men for whom war had long furnished the necessaries of life. So all the Goths approached their king Thiudimer and, with great outcry, begged him to lead forth his army in whatsoever direction he might wish." (Jordanes LVI: 133)

Migration Period (500 - 700 A.D.)

Following Theodoric's conquering of Italy, Clovis I (466 - 511 A.D.) of the Salian Franks would join confederations of other Germanic groups and attack the Visigothic kingdom, defeating them in 507 A.D., cementing Frankish rule over the former province of Gaul by 509 A.D.. The Gepids, the successors to the Huns, would be defeated in 576 A.D. by the Turckic-speaking Avars and the Germanic-speaking Lombards.

The largest population movements in this period occurred outside the scope of written history. The most significant and long-lasting were the movements of Slavic-speaking groups following the Huns and Avars. They settled areas abandoned by the Goths in their conquest of the Roman Empire. The Byzantine Empire would record increasing concentrations of Slavic groups at their borders following 600 A.D. By this time they occupied a region which stretched from the Baltic to the Eastern Alps. Primary historical accounts do not begin again until the 19th century, by which time Slavic-speaking groups were in place in the Balkans and in north-central Europe.

Contemporaneous with these movements, though less well-documented either historically or archaeologically, was the movement of Angle and Saxon tribes into Britain following the power vacuum left in the Roman Empire's wake. This movement would erase Latin as a language and replace it with a West Germanic language that would eventually evolve into English.

In Romania, a population of Latin speakers called the Vlach migrated into what was once Dacia, a Roman province. The origin of this group is unknown - some scholars have argued that they were latinized Dacians who preserved the language following Roman abandonment of the region in the third century A.D. (25), while others argue they were Romans in the Balkans who migrated north during the Migration Period (26). Modern Romanians themselves do not have any legends or cultural affinities with ancient Roman culture beyond language, and while the majority currently reside in Romania many smaller communities are spread out throughout the Balkans.

The movements of this period would ultimately be the seeds of the modern European states. The majority of movements are difficult to infer from either history or archaeology. The continuation of these movements beyond the collapse of the Western Roman Empire suggests that the collapse itself was a symptom of broader changes in the European sub-continent.

Supplemental Figures

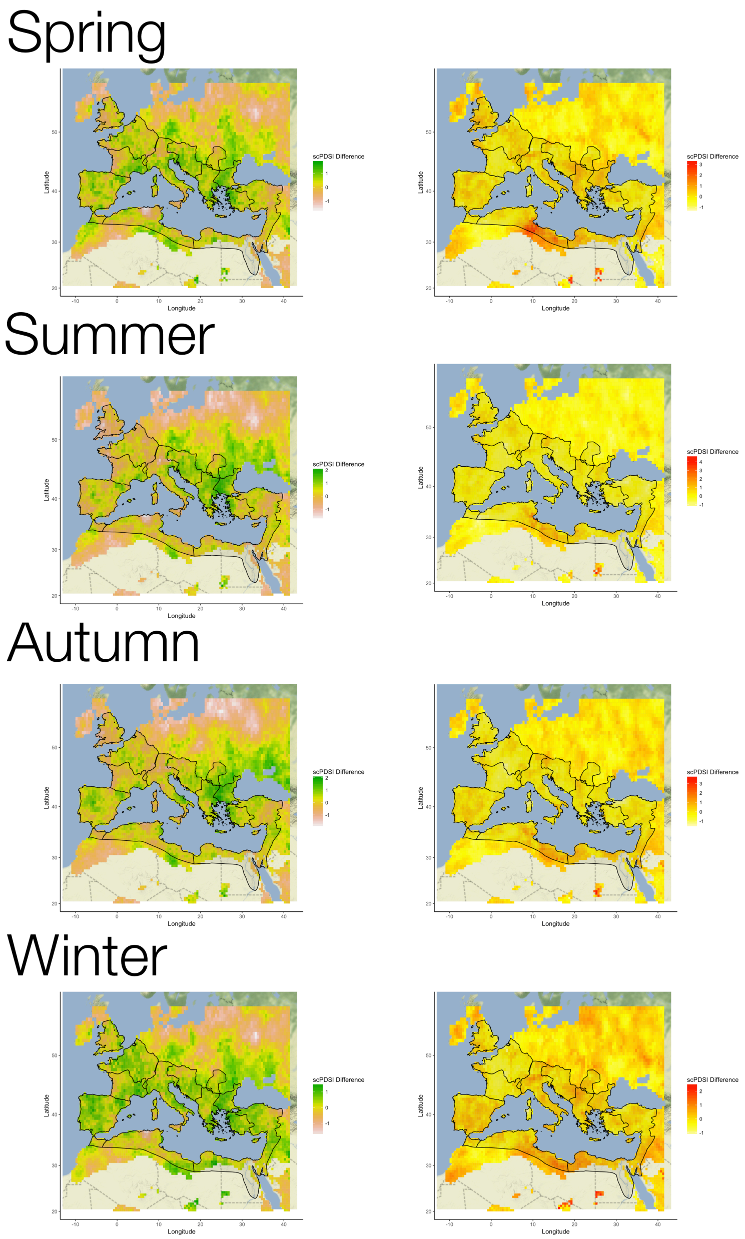

Supplemental Figure 1: Seasonal changes in scPDSI from NAO 1-2 to NAO 0-1, with standard deviations in the records. Map generated in R (3.3.2)(26) using map tiles by Stamen Design (under CC BY 3.0. Data by OpenStreetMap, under ODbL) and historic scPDSI records (27,28).

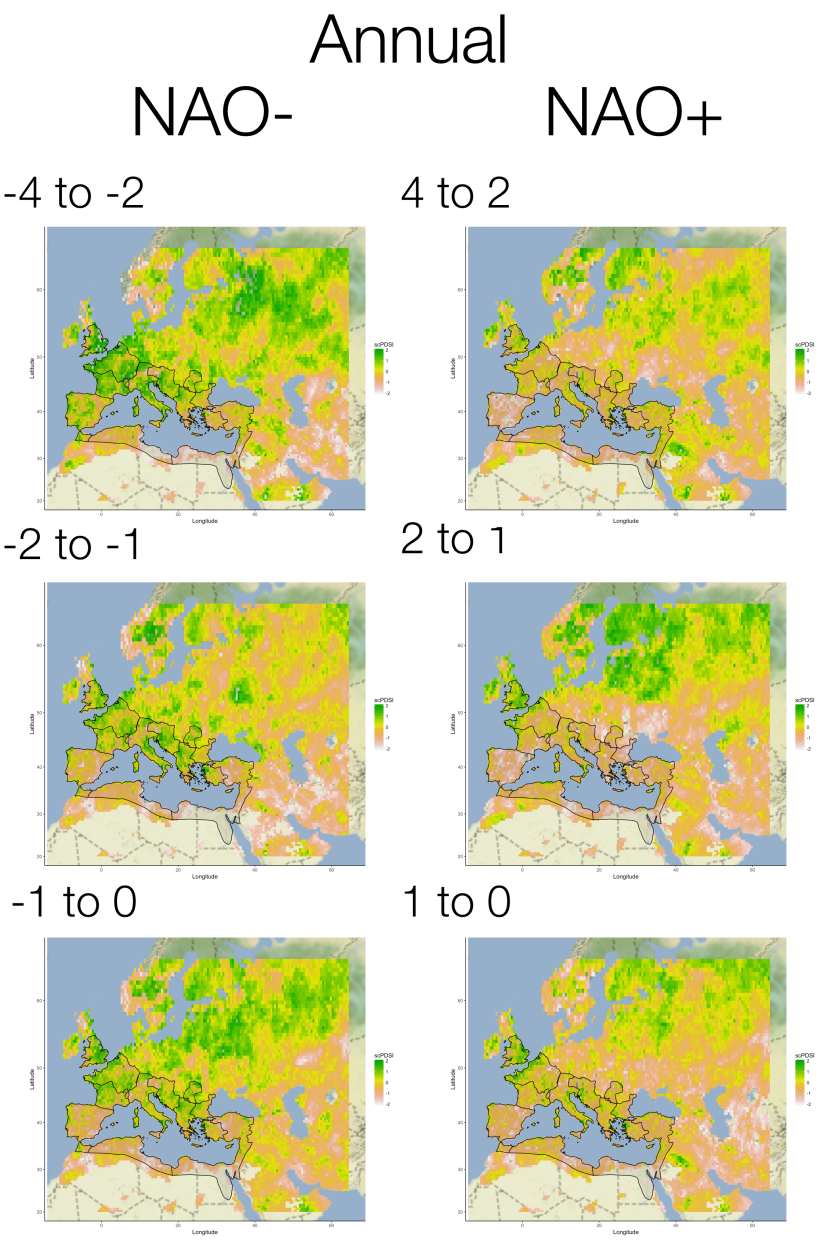

Supplemental Figure 2: Annual changes in scPDSI for different ranges of NAO variation. Map generated in R (3.3.2)(26) using map tiles by Stamen Design (under CC BY 3.0. Data by OpenStreetMap, under ODbL) and historic scPDSI records (27,28).

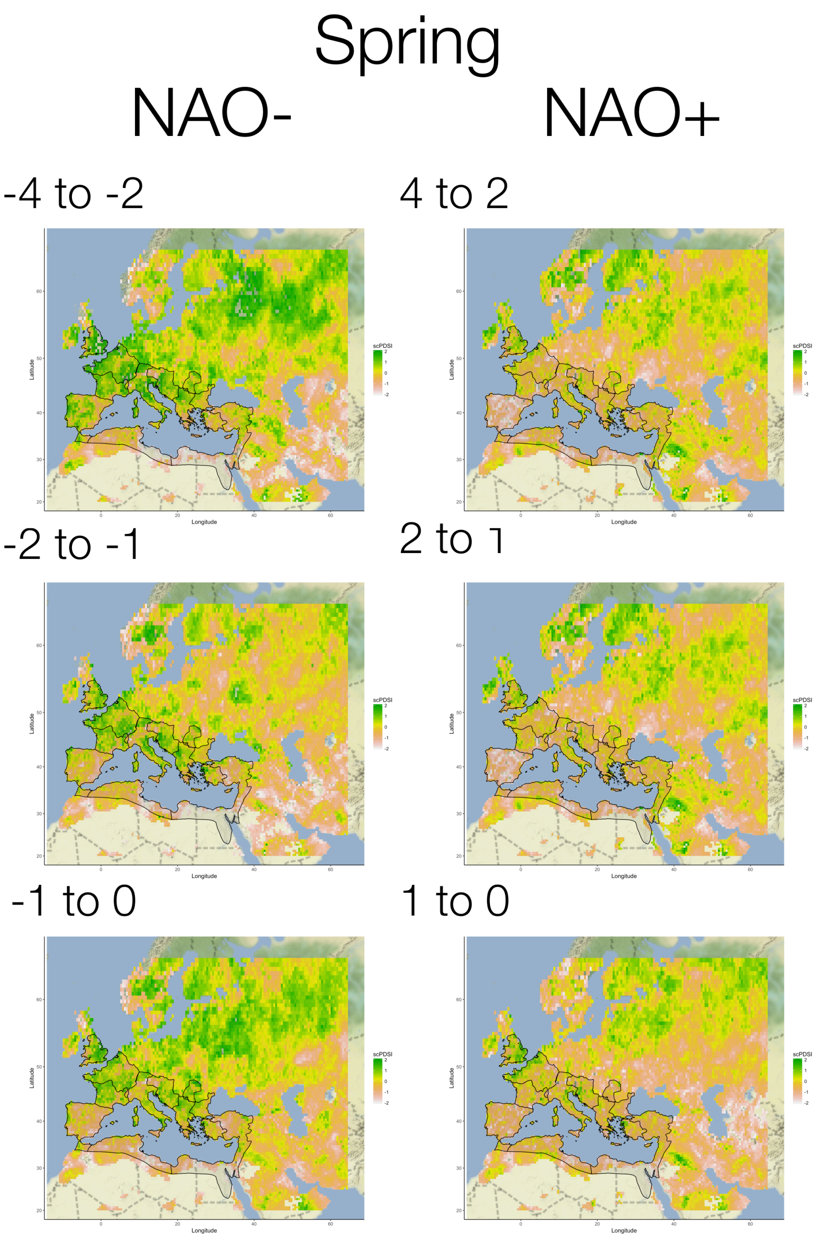

Supplemental Figure 3: Spring (March - May) changes in scPDSI for different ranges of NAO variation. Map generated in R (3.3.2)(26) using map tiles by Stamen Design (under CC BY 3.0. Data by OpenStreetMap, under ODbL) and historic scPDSI records (27,28).

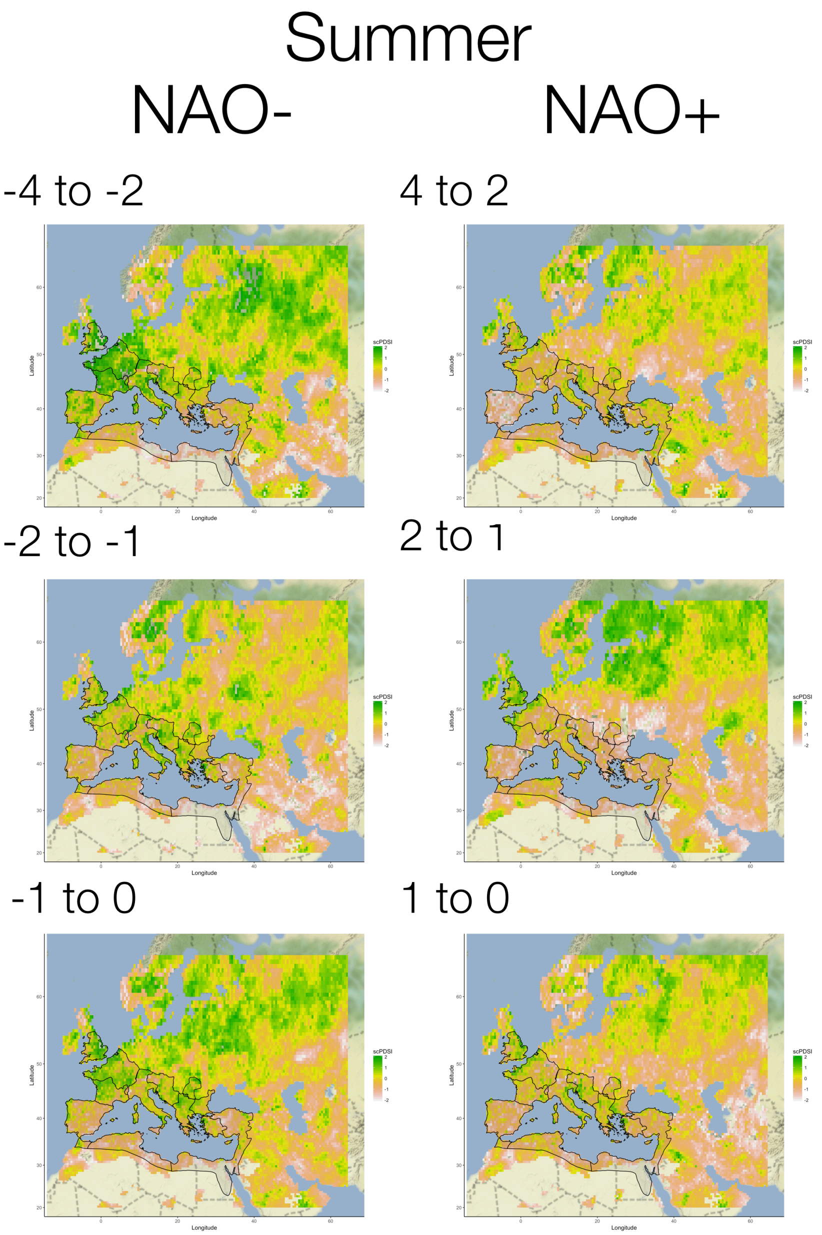

Supplemental Figure 4: Summer (June - August) changes in scPDSI for different ranges of NAO variation. Map generated in R (3.3.2)(26) using map tiles by Stamen Design (under CC BY 3.0. Data by OpenStreetMap, under ODbL) and historic scPDSI records (27,28).

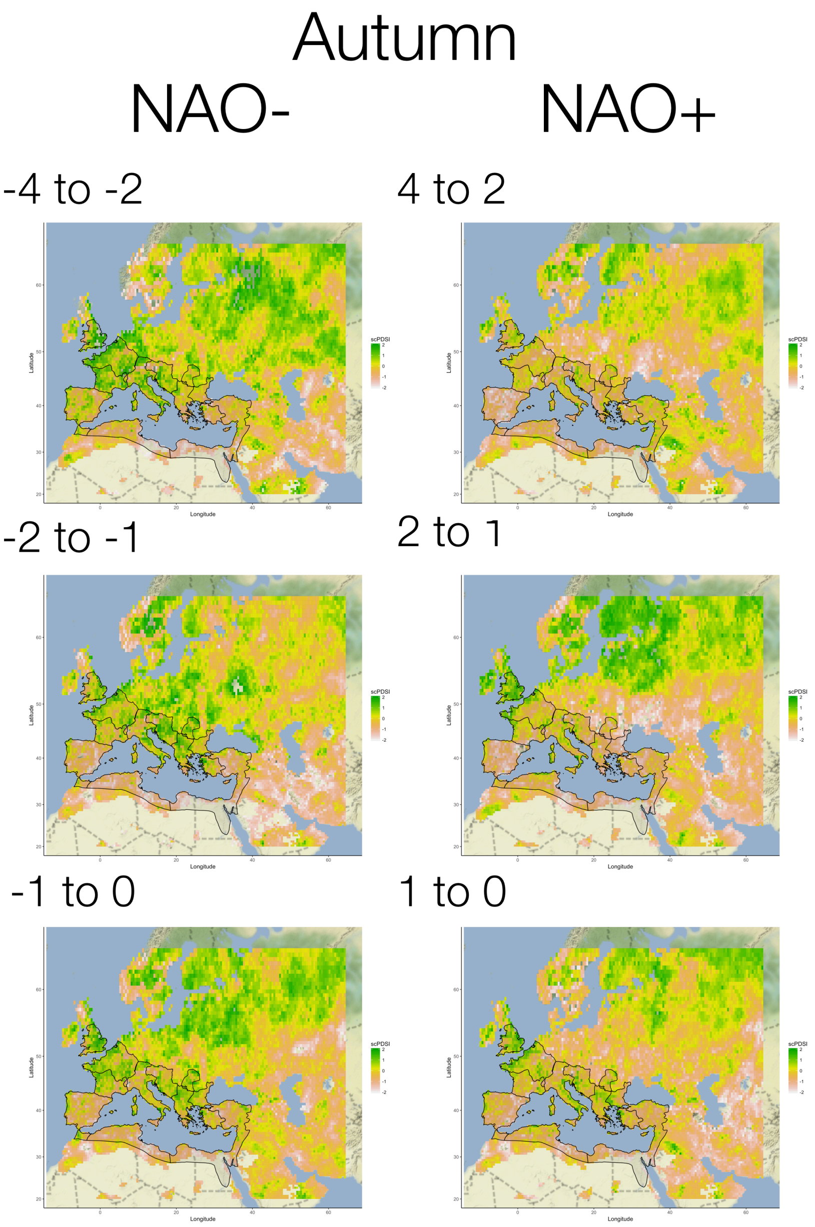

Supplemental Figure 5: Autumn (September - November) changes in scPDSI for different ranges of NAO variation. Map generated in R (3.3.2)(26) using map tiles by Stamen Design (under CC BY 3.0. Data by OpenStreetMap, under ODbL) and historic scPDSI records (27,28).

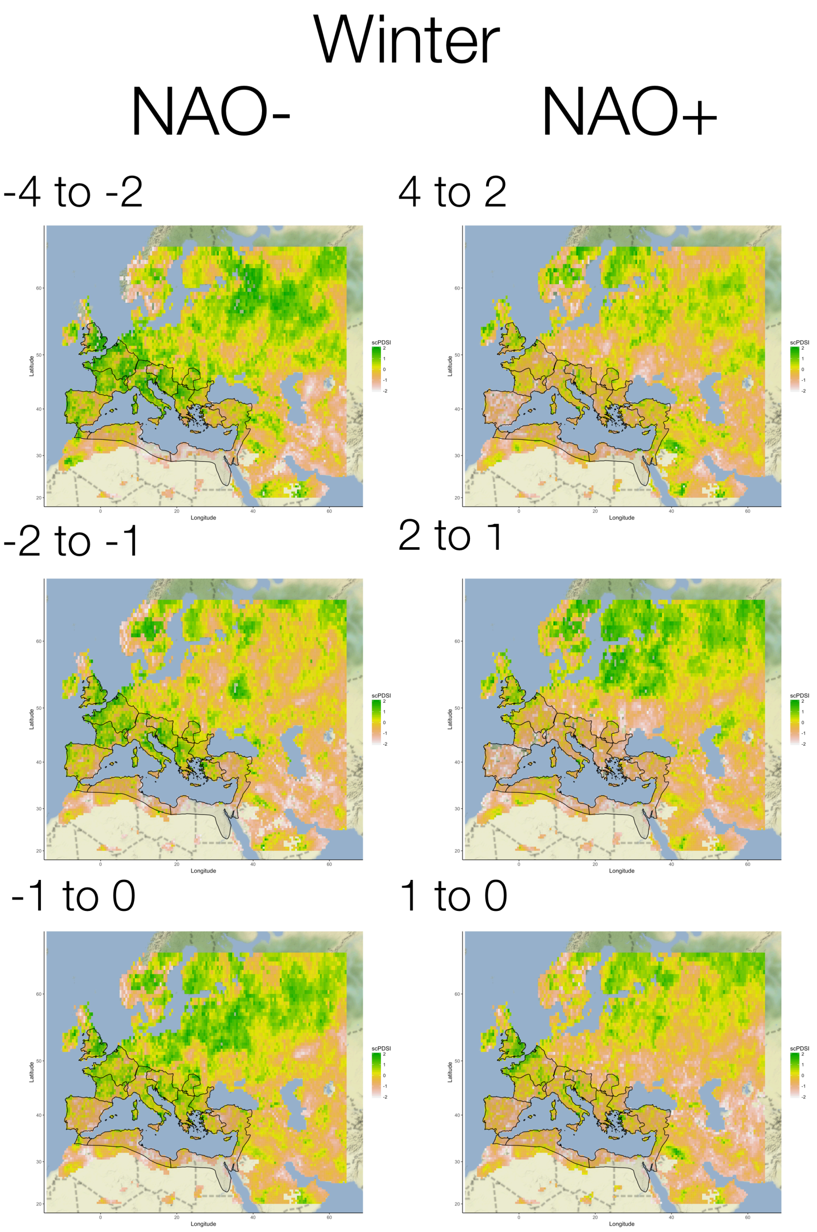

Supplemental Figure 6: Winter (December - February) changes in scPDSI for different ranges of NAO variation. Map generated in R (3.3.2)(26) using map tiles by Stamen Design (under CC BY 3.0. Data by OpenStreetMap, under ODbL) and historic scPDSI records (27,28).

References

1) Baker, G.A.M. *Titus Livius: The History of Rome, Volume VI: Lost Books* 313 - 315 (Peter A. Meiser and Co. 1823).
2) Frankenstein, S., Rowlands, M.J. The internal structure and regional context of of early Iron age society in southwestern Germany. *Bulletin of the Institute of Archaeology* 15: 73-112 (1978).
3) Mauss, M. *The Gift. The Form and Reason for Exchange in Archaic Societies* (W.D. Halls 1950)
4) Haverfield F. *The Romanization of Roman Britain* (Oxford University Press 1912)
5) Hamilton, H.C., Falconer, W. *The Geography of Strabo, Book VII* 443 - 452 (George Bell & Sons 1892)
6) Perrin, B. *Plutcarch Lives IX* 465 - 599 (Harvard University Press 1920)
7) Shipley, F.W. *Velleius Patercullus Compendium of Roman History, Res Gestae Divi Agusti* 345 - 405 (Harvard University Press 1911)
8) Killgrove, K., Montgomery, J. All Roads Lead to Rome: Exploring Human Migration to the Eternal City through Biochemistry of Skeletons from Two Imperial- Era Cemeteries (1st-3rd c AD). *PLoS One* 11(2): e0147585 10.1371/journal. pone.0147585 (2016).
9) Peterson, W.. *Tacitus - Dialogus, Agricola, Germania* 264 - 354 (Harvard University Press 1914).
10) Edwards, H.J.. *Caesar: The Gallic Wars* 344 - 349 (GP Putnam & Sons 1919)
11) Andrzejowska, A. The Przeworsk Culture. A Brief Story (for the Foreigners), In: U. Lund Hansen & A. Bitner-Wróblewska (eds.), *Worlds Apart? Contacts across the Baltic Sea in the Iron Age 1 - 52* (Nordiske Fortidsminder C/7 2010).
12) Mierow, C.C. *The Gothic History of Jordanes* 51 - 142 (Princeton University Press 1915)
13) Zagórska-Telega, J. Some remarks on the funerary rite of the Przeworsk culture in the Younger and Late Roman Periods. *Barbaricum* (8): 263-278 (2009).
14) Cieśliński, A. The presence of flat graves at the burial mound cemeteries of the Wielbark culture in northeastern and eastern Poland. *Wiadomości Archeologiczne*, t. LXIV: 49-235 (2013).
15) Heather, P. Empires and Barbarians: *Migration, Development and the Birth of Europe*. (Pan Macmillian 2010)
16) Cary, E. *Dio's Roman History IX* 2-121 (Harvard University Press 1955)
17) Yonge, C.D. *The Roman History of Ammianus Marcellinus, During the Reigns of the Emperors Constantius, Julian, Jovianus, Valentian, and Valens* 575-623 (George Bell & Sons 1894)
18) Adams, G.W. *Marcus Aurelius in the Historia Augusta and Beyond* 182 - 183 (Lexington Books 2012)
19) Cousin, L. *Histoire des Romans ecrit par Xiphilin, par Zonare, et par Zosime* 485-492 (Verlag 1686)
20) Stanev, A. Observations on the Barbarian presence in the province of Moesia Secunda in Late Antiquity (eds. Stoyan Vitlyanov and Ivo Topalivov) *The Empire and Barbarians in South-eastern Europe in Late Antiquity and the Early Medieval Ages*. Studia Academica Šumenensia I. The University of Shumen Press. ISSN 2367-5446. pp. 65-86 (2014)
21) Sharov, O.V. Early Phase of Chernyakhov Culture (eds. Sharov, O.V., Kazansky, M.) *Saint-Petersburg Apocrypha. Epistle of Mark*) 321 - 340 ISBN 978-9975-4079-3-9 (Stratum 2011).
22) Piontek, Iwanek, B., Segeda, S., Nowak, O. Odontological analysis of central European populations from the Roman period and early middle ages. *Humanbiology Budapest* 30: 77-86 (2007).
23) Vossius, G.J. *The History of Count Zosimus Sometime Advocate and Chancellor of the Roman Empire* 136 - 179 (J. Davis 1814)
24) Thompson, E.A. *The Huns* ISBN 978-0-631-21443-4 (Wiley-Blackwell 1948)
25) Treptow, K.W. Popa, M. *Historical Dictionary of Romania*. ISBN 0-8108-3179-1 (Scarecrow Press, Inc. 1996).
26) Schramm, Gottfried. *Ein Damm bricht. Die römische Donaugrenze und die Invasionen des 5-7. Jahrhunderts in Lichte der Namen und Wörter.* R. Oldenbourg. ISBN 3-486-56262-2. (Verlag 1997)
27) R Core Team. *R: A Language and Environment For Statistical Computing* https://www.t-project.org (R Foundation for Statistical Computing 2017).
28) van der Schrier G., Barichivich J., Briffa K.R. and Jones P.D. A scPDSI-based global data set of dry and wet spells for 1901-2009. *J. Geophys. Res. Atmos.* 118, 4025-4048 10.1002/jgrd.50355 (2013).

29) Osborn T.J., Barichivich J., Harris I., van der Schrier G., Jones P.D. Monitoring global drought using the self-calibrating Palmer Drought Severity Index State of the Climate in 2015. *Bulletin of the American Meteorological Society* 97, S32-S36 (2016).
